# Thyroglossal Duct Papillary Thyroid Carcinoma and Synchronous Lingual Thyroid Atypia

**DOI:** 10.1155/2016/3975924

**Published:** 2016-03-28

**Authors:** Timothy Yoo, Yohanan Kim, Alfred Simental, Jared C. Inman

**Affiliations:** ^1^Loma Linda University School of Medicine, Loma Linda, CA 92354, USA; ^2^Department of Otolaryngology-Head and Neck Surgery, Loma Linda University Medical Center, Loma Linda, CA 92354, USA

## Abstract

Thyroglossal duct and lingual thyroid ectopic lesions are exceedingly rare synchronous findings. Papillary thyroid carcinoma of these ectopic thyroid sites is well understood but still a rare finding. This case points to some management nuances in regard to ectopic thyroid screening with imaging and also shows the effectiveness of minimally invasive transoral robotic surgery for lingual thyroid.

## 1. Introduction

Thyroid dysgenesis is a relatively common phenomenon that can manifest in several ways, including ectopic thyroid tissue and thyroglossal duct cysts. Ectopic thyroid tissue arises from aberrancies in the embryologic descent of the thyroid anlage, resulting in the existence of thyroid tissue outside the normal thyroid compartment. Thyroglossal duct cysts arise from a failure of the thyroglossal duct to involute with subsequent cyst formation via an unknown etiology. Lingual thyroid arises when the thyroid fails to descend and develops in the tongue. The combination of these two abnormal thyroid developments occurring synchronously is exceedingly rare, possibly due to the embryogenesis of the thyroglossal duct not likely progressing to allow thyroid tissue remnants if the lingual thyroid never descends. Subsequent malignant transformation of ectopic thyroid tissue occurs in <1% of cases. In this case report, we present a 41-year-old Hispanic woman's thyroglossal duct cyst with papillary thyroid carcinoma and synchronous lingual thyroid with atypia and multiple foci suspicious for papillary thyroid carcinoma.

## 2. Case Presentation

A 41-year-old Hispanic female had noted a small midline neck mass and was seen by an outside endocrinologist, who ordered a thyroid ultrasound that demonstrated a 25 × 6 × 14 mm left thyroid lobe with 8 and 11 mm nodules as well as a 36 × 10 × 15 mm right thyroid lobe. She stated that she thought the mass had been there since she was young; however, she recently began to feel a globus sensation with occasional dysphagia. She denied a family history of thyroid cancer or irradiation. She was followed up by endocrinology and started on levothyroxine 50 mcg/day. Over the next 2 years, the patient noticed the mass enlarging and the globus-like feeling worsening and represented to an outside facility for a repeat thyroid ultrasound, which showed a 19 × 6 × 7 mm left thyroid lobe and 19 × 6 × 5 mm right thyroid lobe along with a prominent 34 × 26 × 37 mm midline neck mass. Labs showed thyroid stimulating hormone (TSH) level of 3.890, free T4 level of 11.1, thyroid peroxidase antibody level of 6, thyroglobulin antibody level <1, and a calcium level of 9.6.

The patient was diagnosed with a thyroglossal duct cyst and was referred for a fine-needle aspiration (FNA) and a surgical consultation. Thyroglossal duct cyst FNA showed no malignant cells. Surgical excision of the cyst was performed transcervically with a Sistrunk procedure, removing a 40 × 36 × 30 mm mass. Final pathology showed 3 separate foci of papillary thyroid carcinoma that were 4 mm, 5 mm, and 5 mm in diameter. Both classic papillary and follicular variants were present. These microcancers were well differentiated, partially encapsulated, and had clear margins; no extrathyroidal, perineural, or lymphovascular spread was noted. Pathologic staging indicated ectopic multifocal T1aNxMx papillary thyroid carcinoma. MACIS score was calculated to be 3.43.

When questioned about these outside institution ultrasound report discrepancies, the patient stated that both ultrasound experiences were “odd” and that they both took “hours” to perform as the original ultrasound technician had difficulty locating her thyroid. According to the patient, multiple technicians and a radiologist had to be consulted before a thyroid was “found and measured” as documented in their report. One technician told her she had an atrophic thyroid gland that was smaller than expected. The patient was scheduled for a CT neck scan after noting the discrepancies in her thyroid ultrasound reports and in preparation for determining how to follow up her native thyroid gland.

**Figure 1:**
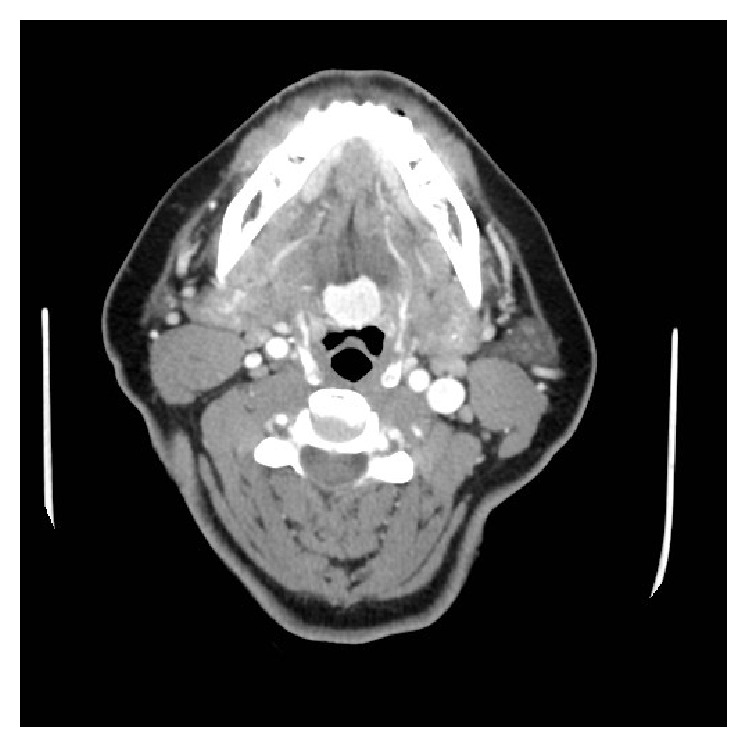
Axial CT neck showing a lingual thyroid.

**Figure 2:**
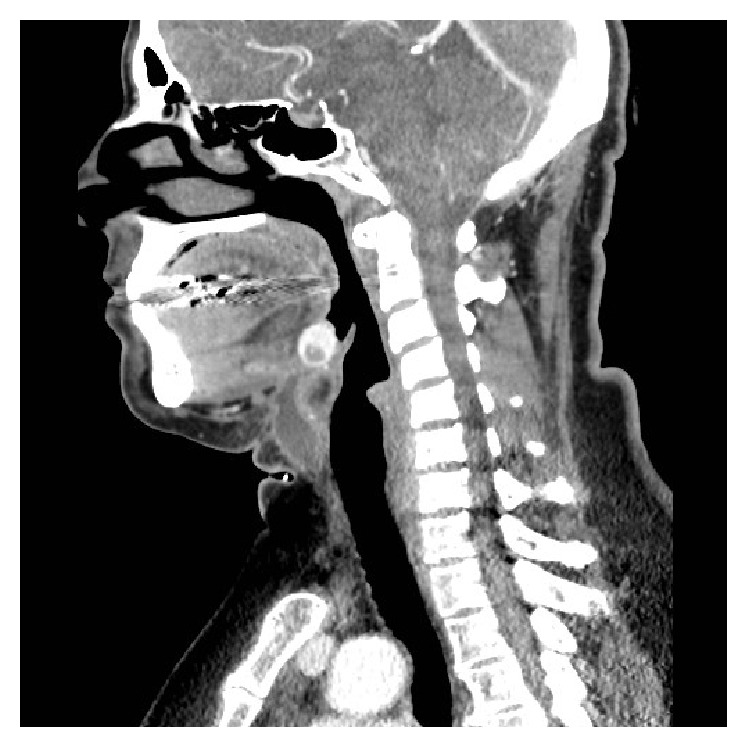
Sagittal CT neck revealing a lingual thyroid without a thyroid in its native position.

The CT scan showed a 26 × 28 mm mass in the tongue base with hypodense nodules up to 7 mm in diameter (Figures 1 and 2). A thyroid gland in its native position was not appreciated (Figure 2). An intrainstitutional ultrasound showed a 2.0 × 1.6 × 1.4 cm tongue base mass with microcalcifications and internal vascularity; no normal thyroid tissue was present in the neck or thyroid bed.

The patient was counseled extensively on the treatment of micropapillary thyroid carcinoma, including the need for close observation, radioactive iodine, and potentially the removal of the thyroid tissue. The need for serial blood work, follow-up scans, and TSH suppression via levothyroxine were also explained to the patient. She also had concerns regarding her globus sensation and dysphagia, which had not resolved and had been worsening. In-office flexible endoscopy and CT scan both showed a submucosal fullness in the vallecula obliterating the space between the base of tongue and the epiglottis. The patient had also been on levothyroxine for three years prior to surgical intervention for thyroid dysfunction. Due to the multifocality of her ectopic thyroid tissue, the microcalcifications present in the lingual thyroid, and her distrust in the monitoring process due to her previous experiences with thyroid ultrasounds, she elected for lingual thyroid removal.

**Figure 3:**
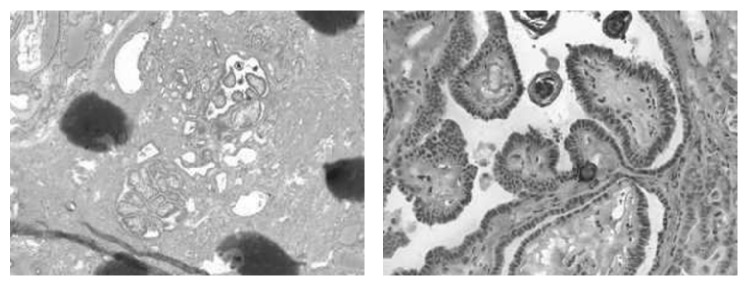
Histopathology of lingual thyroid tissue demonstrating papillary thyroid carcinoma.

Lingual thyroid excision via transoral robotic surgery (TORS) was performed in May 2014, lasting 37 minutes without complications. The patient stayed in the hospital one night and was discharged on a liquid diet. Pathology demonstrated a 29 × 27 × 20 mm base of tongue mass showing thyroid tissue with focal atypia suspicious for papillary thyroid carcinoma at three separate foci with nuclear enlargement, focal nuclear membrane irregularity, and some nuclear grooves (Figure 3); microcalcifications were present. Postoperative recovery was in line with our transoral base of tongue surgery experience, with patient follow-up at 1 and 3 weeks postoperatively and with return to normal diet by week 3. She has continued to be followed up by our institution since discharge. Flexible endoscopy showed a well-mucosalized tongue base and a 6-month postexcision CT scan for baseline anatomy for long-term surveillance showed no residual abnormalities in the base of the tongue or neck. TSH suppression via levothyroxine has remained adequate, with the most recent TSH level measuring less than <0.5 mU/L, and serum thyroglobulin has remained negative. She has not required irradiation. I-131 scintigraphy and thyroglobulin tumor markers show no evidence of recurrence to date.

## 3. Discussion

Thyroid tissue initially forms via the differentiation of endodermal epithelial cells at the foramen cecum of the tongue of the developing fetus. During the fourth week, a diverticulum formed from the 1st and 2nd pharyngeal pouches (the thyroid anlage) descends along the midline neck down to the position of the normal thyroid while maintaining a narrow connection with the foramen cecum, otherwise known as the thyroglossal duct. The descent and differentiation of the thyroid lobes are complete by the seventh week and are followed by atrophy of the thyroglossal duct by the tenth week.

Ectopic thyroid tissue arises as a consequence of its migration to the neck during embryogenesis. As the thyroid cells descend, they may be stopped or trapped along the migration path. This most frequently occurs along the thyroglossal duct but can rarely arise in more remote locations. Failure of thyroid gland descent occurs in 1/200,000 normal subjects and 1/6000 subjects with known thyroid disease [1]. Defects can also arise from failure of the thyroglossal duct to atrophy, resulting in the formation of cysts. Of these defects, lingual thyroid and thyroglossal duct cysts are the most common; the prevalence of lingual thyroid in the general population is estimated to be between 1 : 100,000 and 1 : 300,000 [2], while the incidence of thyroglossal duct cysts in the general population is approximately 7% [3]. Furthermore, it is estimated that lingual thyroids form 90% of ectopic thyroid cases [4]. Despite the prevalence of the deformities themselves, the incidence of both lingual thyroid and thyroglossal duct cyst occurring concurrently is exceedingly rare. A case report by Madana et al. in 2012 found only one previous report of such scenario in the literature before their own [5]. To our knowledge, the exact embryogenesis of lingual thyroid and thyroglossal duct cysts as synchronous lesions is unknown; however, it is possibly simply explained by partial descent with arrested cell growth in multiple areas.

There is a very small risk of malignant transformation associated with these ectopic thyroid malformations. Approximately 1% of patients with thyroglossal duct cysts demonstrate malignant transformation [6] and less than 1% of lingual thyroids undergo malignant transformation [2]. The incidence of multiple foci of cancerous ectopic thyroid tissue (nonmetastatic) is likely even more rare as we could not locate any such cases. It is theorized that these malignancies demonstrate either a de novo mutation or a metastasis from an occult primary thyroid gland [7, 8]. Our patient demonstrated papillary microcarcinomas or papillary carcinomas measuring less than or equal to 1 cm [9]. This is a relatively common finding in patients with thyroid cancer and one that must be considered in all cases. In a retrospective case study, Pakdaman et al. found papillary microcarcinomas in nearly 50% of 860 cases of patients with papillary thyroid carcinoma [10].

The formation of papillary thyroid carcinoma within thyroglossal duct cysts, however, is much rarer. A review of the literature suggests the rate of thyroglossal duct cyst carcinomas (TGDCC) to be 1-2% [11, 12]. Diagnosis is difficult as differentiating TGDCCs from their benign counterparts cannot be confirmed without histopathologic examination of the cyst itself; imaging and fine-needle aspiration (FNA) are unreliable measures, with FNA being correct only 53–66% of the time [13]. As such, malignancy of the thyroglossal duct cyst may not be suspected until removal and examination of the cyst. Prognosis of these malignancies, however, is excellent with less than 2% demonstrating metastasis [14]. Though there is no official consensus nor protocol regarding the treatment of TGDCCs, the Sistrunk operation appears to be the most commonly used technique for the resection of the cyst [11–14].

As our case demonstrates, the detection of ectopic thyroid tissue can be difficult and may require multiple imaging modalities. Ultrasonography (US) is an attractive screening option given its relatively low cost, speed, and ease of use. However, its effectiveness in ectopic thyroid tissue sensitivity or specificity is questionable. Jain et al. noted a very low detection rate with 0–21% accuracy in detecting ectopic thyroid tissue via ultrasound in their review of pediatric ectopic thyroid cases [15]. Furthermore, a study comparing the reliability of thyroid scintigraphy to thyroid ultrasonography in 82 children with previously diagnosed thyroid dysgenesis noted a sensitivity and specificity of 10 and 100 in detecting ectopic tissue with US versus 92 and 97.1 with scintigraphy [16]. Our case is in line with these studies as our patient's multiple foci of ectopic thyroid tissue were either missed, inaccurately measured by US, or recorded as normal thyroid in the thyroid bed when none was actually present. As such, the use of multiple imaging modalities may be prudent in ectopic thyroid cases.

Medical management is recommended in most cases of asymptomatic lingual thyroid, often by thyroid suppression therapy via administration of exogenous thyroid hormone [17]. Other treatment modalities include radioactive iodine treatment or surgery. In our case, the patient opted for surgical excision via TORS due to concern for lingual thyroid malignancy, her preexisting globus, and her levothyroxine use from thyroid dysfunction. Primary modalities for lingual thyroid excision historically are invasive open approaches including the transcervical pharyngotomy approach, the midline labiotomy, and/or mandibulotomy for surgical access. However, these procedures are linked with high morbidity due to the invasive nature of these approaches, long operation times, prolonged hospitalization, and cosmetic scarring [17]. Transoral resections have also been previously reported. These cases can be technically difficult to teach young surgeons due to limited perspective, scope exposure, patient anatomic restrictions, and poor surgical access to the tongue base deep in the vallecula where most lingual thyroids sit deep to the lingual tonsil [18]. In our experience with transoral resections since the development of new instrumentation, specifically the Hinni modified Lindholm distending laryngoscope and the hand held laser (rather than the line of site), these bases of tongue resections have become technically more attainable and teachable. However, the superior anterior vector of dissection with the scope in place for the deep vallecula is still challenging. We have noted that the lingual thyroid tissue is highly vascularized and staying outside the “capsule” or maintaining a small cuff of muscle and lingual tonsil subjectively seems to bleed less and provide better quality tissue for grasping and retracting—something that is crucial for deep base of tongue surgery performed transorally. The continued development of minimal TORS overcomes some of these limitations by providing 3D visualization and robotic instrumentation allowing for more angles of dissection. The articulating arms with greater degrees of freedom and the enhanced video with deeper more angulated viewpoints may allow better access to the deep vallecula in some surgeons hands. A literature review by our authors demonstrated 6 case studies/series with a total of 12 patients who underwent TORS for lingual thyroid resection. All studies suggested that a TORS approach will likely decrease morbidity, operation duration, and hospital stay length in these patients compared to more traditional surgical methods [17–22]. Given these findings, and our experience, we believe TORS to be a safe and viable alternative to current surgical interventions for lingual thyroid, where thyroid carcinoma and dissection margin is of importance for complete excision. As always, patients with ectopic thyroid tissue should be screened carefully and minimally invasive procedures, when indicated for thyroid tissue removal, can aid patients in avoiding some morbidity.

## 4. Conclusion

Detection and treatment of ectopic thyroid tissue and the exceedingly rare thyroid cancer which may be concurrent are topics which occasionally are in need of reevaluation. Recommendations regarding detection of ectopic thyroid tissue and current screening regimens may not be sufficient to detect all incidences of ectopic thyroid when the accuracy of such testing is not well understood and may change treatment. In our patient, the inaccurate US report exposed the patient to an extraoperative encounter. We attribute this inaccuracy to technician error, possibly arising from inexperience or preconceived expectations on the part of the interpreter. As such, we recommend utilizing multiple imaging modalities in rare cases of ectopic thyroid malignancy. Preoperative scintigraphy or CT scan can aid in detection of ectopic thyroid. In addition, studies with more rigorous methodologies to evaluate new surgical techniques used in treating ectopic thyroid tissue, specifically in the base of tongue, are necessary given the rise of novel tools such as the handheld laser fiber and TORS.
